# Black coral forests enhance taxonomic and functional distinctiveness of mesophotic fishes in an oceanic island: implications for biodiversity conservation

**DOI:** 10.1038/s41598-023-32138-x

**Published:** 2023-03-27

**Authors:** Nestor E. Bosch, Fernando Espino, Fernando Tuya, Ricardo Haroun, Lorenzo Bramanti, Francisco Otero-Ferrer

**Affiliations:** 1 Asociación Biodiversidad Atlántica y Sostenibilidad (ABAS), 35214 Telde, Spain; 2 Grupo en Biodiversidad y Conservación (IU-ECOAQUA), Universidad de Las Palmas de Gran Canaria, 35214 Telde, Spain; 3 Sorbonne Université, CNRS, Laboratoire d’Ecogéochimie des Environnements Benthiques, LECOB, 66500 Banyuls-sur-Mer, France

**Keywords:** Biodiversity, Community ecology, Conservation biology

## Abstract

The degradation of shallow ecosystems has called for efforts to understand the biodiversity and functioning of Mesophotic Ecosystems. However, most empirical studies have been restricted to tropical regions and have majorly focused on taxonomic entities (i.e., species), neglecting important dimensions of biodiversity that influence community assembly and ecosystem functioning. Here, using a subtropical oceanic island in the eastern Atlantic Ocean (Lanzarote, Canary Islands), we investigated variation in (a) alpha and (b) beta functional (i.e., trait) diversity across a depth gradient (0–70 m), as a function of the presence of black coral forests (BCFs, order Antipatharian) in the mesophotic strata, a vulnerable but often overlooked ‘ecosystem engineer’ in regional biodiversity. Despite occupying a similar volume of the functional space (i.e., functional richness) than shallow (< 30 m) reefs, mesophotic fish assemblages inhabiting BCFs differed in their functional structure when accounting for species abundances, with lower evenness and divergence. Similarly, although mesophotic BCFs shared, on average, 90% of the functional entities with shallow reefs, the identity of common and dominant taxonomic and functional entities shifted. Our results suggest BCFs promoted the specialization of reef fishes, likely linked to convergence towards optimal traits to maximize the use of resources and space. Regional biodiversity planning should thus focus on developing specific management and conservation strategies for preserving the unique biodiversity and functionality of mesophotic BCFs.

## Introduction

Uncertainty on the fate of shallow ecosystems under future warming scenarios and increasing human impacts^1,2^ has called for global efforts to expand our knowledge of the biodiversity and functioning of deeper ecosystems. Mesophotic Ecosystems (MEs) are usually found at depths ranging between 30 and 150 m. This depth interval, defined as the mesophotic zone, is characterized by low light penetration, and its limits have been recently suggested to be set between 10 and 1% of the photosynthetically active radiation^3,4^. Declines in the light available for photosynthesis, among other biophysical parameters (e.g., temperature, wave energy), shape patterns of benthic community structure across depth gradients. For instance, in tropical regions, shifts in coral community composition^4^ are often accompanied by a parallel shift in coral morphology^5^ and growth rates^6^, as a result of abiotic constraints. Reduced ecological performance of light-dependent communities (scleractinian corals and macroalgae) often entails a shift in the dominant benthic species to heterotrophic organisms (e.g., sponges and octorals) that are better adapted to low-light conditions^7,8^. Fishes are highly responsive to both changes in abiotic conditions that occur across depth gradients, as well as changes in benthic community structure, particularly specialist species that rely strongly on the habitat and resources provided by foundational species^9,10^. Recent studies on fish assemblages from several euphotic-mesophotic transition zones across various tropical regions (e.g., Caribbean, South-Western and Mid-Atlantic, and Indo-Pacific Ocean) have found high turnover in species composition between shallow and mesophotic depths^11–14^. This singularity in the taxonomic composition of MEs highlights their role as a unique biodiversity element that warrants inclusion in regional conservation planning^15,16^. However, the extent to which this compositional shift occurs in mesophotic ecosystems at higher latitudes such as subtropical, temperate, and polar regions, has received comparatively little scientific attention^17,18^.

At higher latitudes, MEs are often dominated by ecosystem engineers non-dependent on light, such as megabenthic suspension feeders. These communities have been defined as Marine Animal Forests (MAFs) in analogy with terrestrial counterparts with which they share structural and functional similarities, with the difference that MAFs are dominated by animals instead of plants. MAFs represent one of the most widely distributed ecosystems on the planet (e.g., from shallow mussel beds to deep cold-water coral communities) ranging from tropical to polar latitudes^19^. MAFs can shape the seascape by providing a three-dimensional structure, modifying current flow^20^ and light irradiance^21^, and changing the distributional patterns of sediments and particles^22^. Consequently, MAFs mediate biogeochemical cycles in the under-canopy environment^22^ and thus constitute a key habitat offering ecological niches that can host thousands of species^19^. Despite the importance of MAFs as reservoirs of species diversity in coastal ecosystems, we currently have a limited understanding of associated fish communities and the processes involved in community assembly^23^.

The processes involved in community assembly can be inferred from measures of species’ functional traits—e.g., morphological, physiological, and/or behavioral attributes *–* that determine their performance in response to changes in the abiotic environment and biotic interactions^24^. There are precise ecological predictions concerning the importance of abiotic filters (i.e., “Environmental Filtering Hypothesis”) and biotic interactions (i.e., “Competitive Exclusion Hypothesis”) to generate biodiversity patterns across the euphotic-mesophotic zone. Shallow reef areas are less stressful environments, where high energy availability (e.g., higher light irradiance and warmer temperatures) and stronger interspecific interactions might enhance ecological specialization (i.e., trait divergence), minimizing competition for resources (i.e., niche partitioning)^25,26^. In contrast, increased abiotic constraints (i.e. light and temperature reduction) might limit the ecological niches available to reef fishes, driving convergence towards optimal traits (i.e., trait convergence) to maximize the use of resources^27,28^.

Importantly, shifts in the trait composition of reef fishes can have flow-on effects on the movement and storage of energy and materials (i.e., ecosystem functions)^29^. The latter is exemplified in studies on the trophic structure of reef fishes across euphotic-mesophotic zones, where species relying on epibenthic productivity (e.g., herbivorous and invertivorous pathways) tend to dominate shallow reefs, while species depending on planktonic productivity tend to dominate in deeper areas^10,12,30^. Understanding these processes, and the extent to which shallow and mesophotic reefs overlap in their functional (i.e., trait) composition and structure, is thus a critical knowledge gap to understand the extent to which mesophotic reefs can serve as refugia of core ecosystem functions on rapidly eroding shallow reefs^31^.

**Figure 1:**
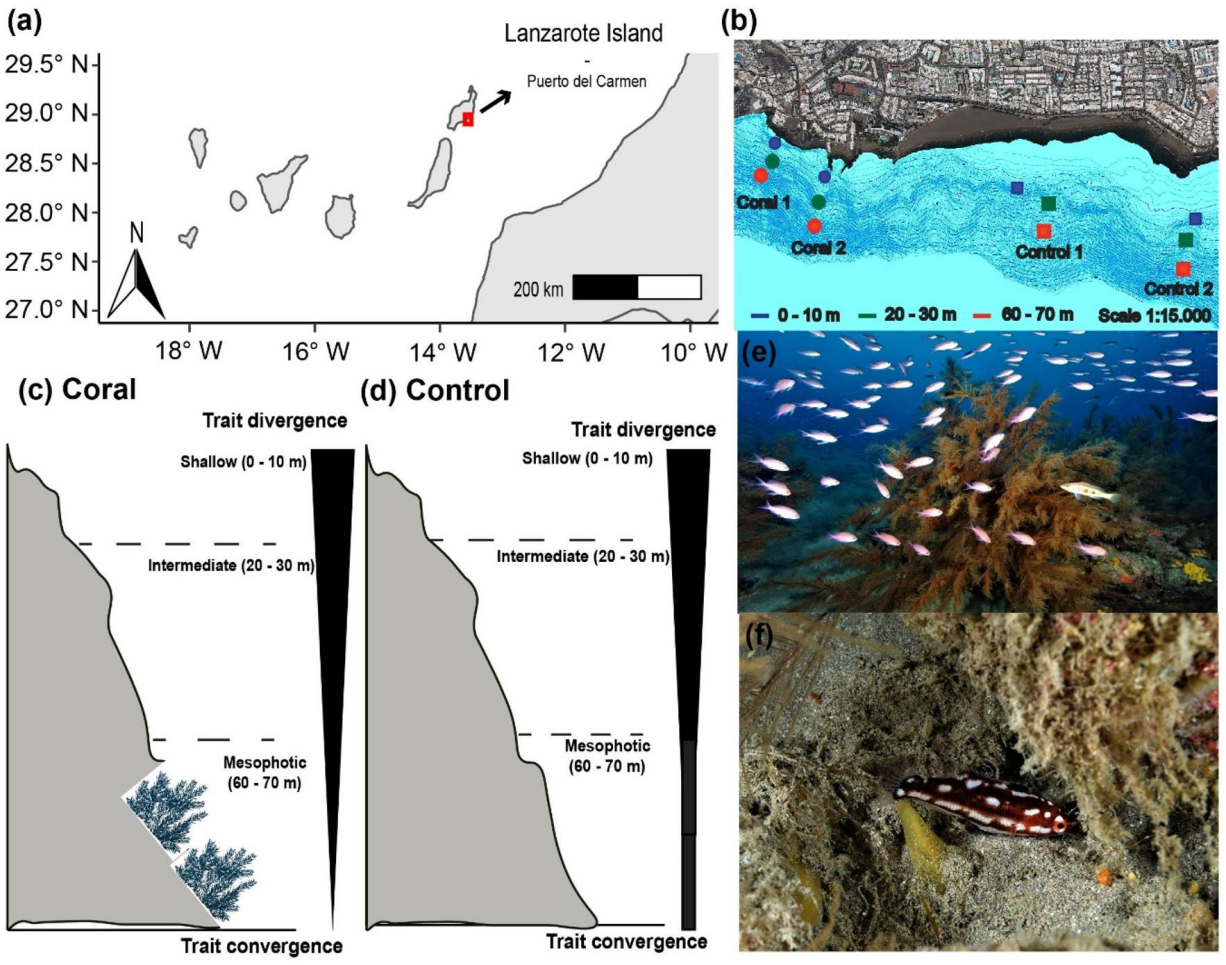
(**a**, **b**) Map of the study area, including the distribution of coral and control sites for each depth strata. (**c**, **d**) Conceptual diagram illustrating changes in the degree of trait divergence *vs.* convergence at coral and control sites. The width of the arrow indicates the magnitude of convergence at the mesophotic strata. (**e**, **f**) Fish assemblages associated with black coral forests in the study region, including (**e**) *Anthias anthias* and *Serranus atricaudata*, and (**f**) *Lapanella fasciata*. Photos: Fernando Espino. (**a**) Map sourced from ‘rnaturalearth’ R package^80^. (**b**) Map sourced from GRAFCAN (https://visor.grafcan.es/visorweb/).

Across many subtropical oceanic archipelagos, MEs are dominated by complex and unknown MAFs structured by Antipatharians (Black Coral Forests, BCFs) as a result of centuries or millennia of biological activity^19^. Despite the importance of BCFs as an ‘ecosystem engineer’ of the circalittoral (ca. 50–200 m) in these regions^32,33^, there is limited information on their associated fish assemblages in mesophotic depths^34,35^. Here, we fill this knowledge gap by testing the role of BCFs in structuring fish assemblages at the (1) taxonomic and (2) functional level across a broad depth gradient (0–70 m) in an oceanic island of the subtropical eastern Atlantic Ocean (Lanzarote Island, Canary Islands, 28° N) (Fig. 1). We first quantified multiple dimensions of alpha functional diversity, capturing the range and variation of functional traits (i.e., richness, evenness, and divergence^36^) to explore the ecological processes that have contributed to the generation and maintenance of fish biodiversity in these unexplored MAFs. Then, we investigated dissimilarities (beta-diversity) in their taxonomic and functional structure to test whether mesophotic BCFs contain unique species and functional roles. This will help us bridge major gaps in MEs and MAFs functioning in subtropical regions, providing key information that can aid the increasing need to protect these unique and valuable ecosystems.

## Results

### Taxonomic structure

A total of 92,972 fishes were recorded belonging to 55 ray-finned species (class Actinopterygii) within 19 orders, 26 families and 47 genera. Species richness declined from the shallow to the mesophotic strata, whilst fish abundance increased, a pattern that was maintained irrespective of the presence of BCFs (Fig. S3).

**Figure 2:**
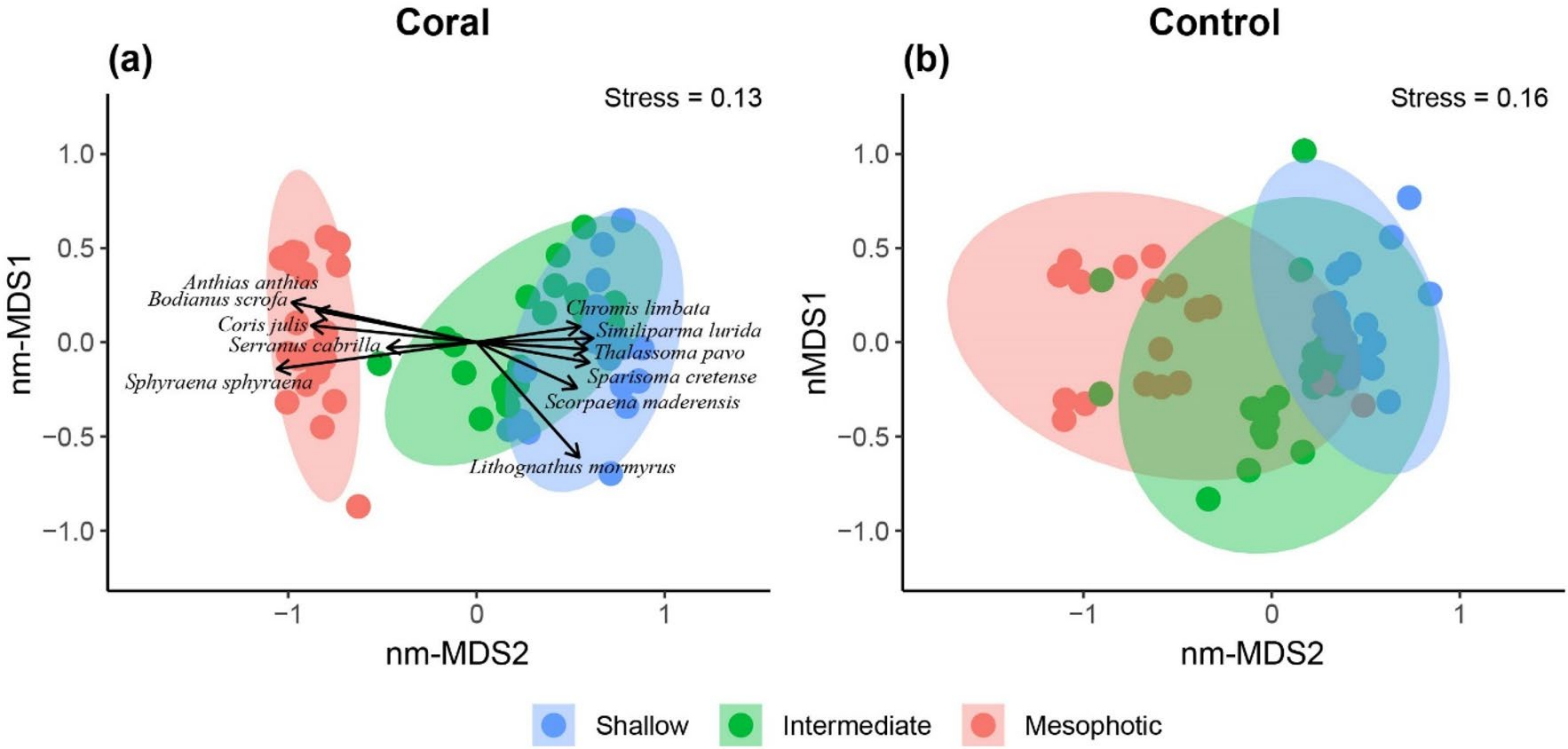
Non-metric multidimensional scaling (nm-MDS) biplot showing the ordination of fish assemblages in the presence or absence of BCFs (a and b, respectively) in 3 different depth strata (shallow, intermediate, and mesophotic). Colored dots depict individual UVCs transects. Confidence ellipses delineate the 95% confidence interval around the mean bivariate coordinate for each depth strata. Species varying significantly among depth strata (Table S4) are overlaid as vectors. Stress values for nm-MDS are indicated within each panel.

Differences in fish assemblage structure (taxonomic level) among depth strata significantly differed between coral and control assemblages (‘depth × habitat’, mvabund, df = 131, *p* = 0.001). Shallow (0–10 m), intermediate (20–30 m), and mesophotic (60–70 m) fish assemblages were separated across the bidimensional ordination space, with moderate overlap of confidence ellipses between shallow and intermediate assemblages, irrespective of the habitat (Fig. 2a, b). Mesophotic fish assemblages in the absence of BCFs displayed higher dispersion around the bivariate mean and moderate levels of overlap with shallow and intermediate assemblages (Fig. 2b). In contrast, mesophotic fish assemblages within BCFs displayed lower dispersion around the bivariate mean and no overlap with shallow and intermediate assemblages (Fig. 2a). Variation in fish assemblage structure was driven mainly by 11 fish species, whose abundances significantly differed among depth strata (multiple GLMs, Table S4). Shallow and intermediate fish assemblages were characterized by higher abundances of the Azores damsel (*Chromis limbata*), the Canary damsel (*Similiparma lurida*), the Ornate wrasse (*Thalassoma pavo*), the Parrotfish *Sparisoma cretense*, the Madeira rockfish (*Scorpaena maderensis*), and the Sand Steenbras (*Lithognathus mormyrus*). Mesophotic fish assemblages associated with BCFs were characterized by higher abundances of the Swallowtail seaperch (*Anthias anthias*), the Barred hogfish (*Bodianus scrofa*), the Mediterranean rainbow wrasse (*Coris julis*), the Comber (*Serranus cabrilla*), and the Yellowmouth barracuda (*Sphyraena viridensis*).

### Functional structure

The 4-D functional space explained 70% of the variation in the traits of the fish species recorded. Most of this variation was accounted for by the first two dimensions, which explained 46% of the variation. The first dimension explained 27% of the variation and mainly separated species based on their home range, group size, and level in the water column (Table S5a). Solitary species with a sedentary behavior living in close association with the bottom, are clustered in the right-hand side of the functional space, while very mobile pelagic species that form large schools clustered in left-hand side (Figs. S5, S7). Fishes were also separated based on their caudal fin shape along this dimension, with species with a rounded or truncated shape clustering in the right-hand side and species with a forked shape clustering in the left-hand side (Figs. S6, S7). The second dimension explained 19% of the variation, and mainly separated species based on their diel activity, preferred temperature, body size and shape, diet, and spawning strategy (Table S5b). Species that clustered in the upper right-hand side of the functional space were generally larger-bodied carnivorous fishes with an elongated or fusiform shape, nocturnal habits, a preference for colder waters, and a pelagic spawning behavior (Figs. S5, S6, S7). In contrast, species that clustered in the lower left-hand side of the functional space were generally smaller-bodied planktivorous or omnivorous fishes with a box or compressed shape, diurnal habits, preference for warmer waters, and a demersal spawning strategy. The third and fourth dimensions explained little additional variation (PCoA3 = 15%, PCoA4 = 7%, Total = 22%), mainly attributed to mouth position (Table S5c,d). Species with superior and subterminal mouth positions clustered in the lower left-hand side, while species with terminal and tubular mouth positions clustered in the upper right-hand (Figs. S6, S8).

### Functional alpha diversity

**Figure 3:**
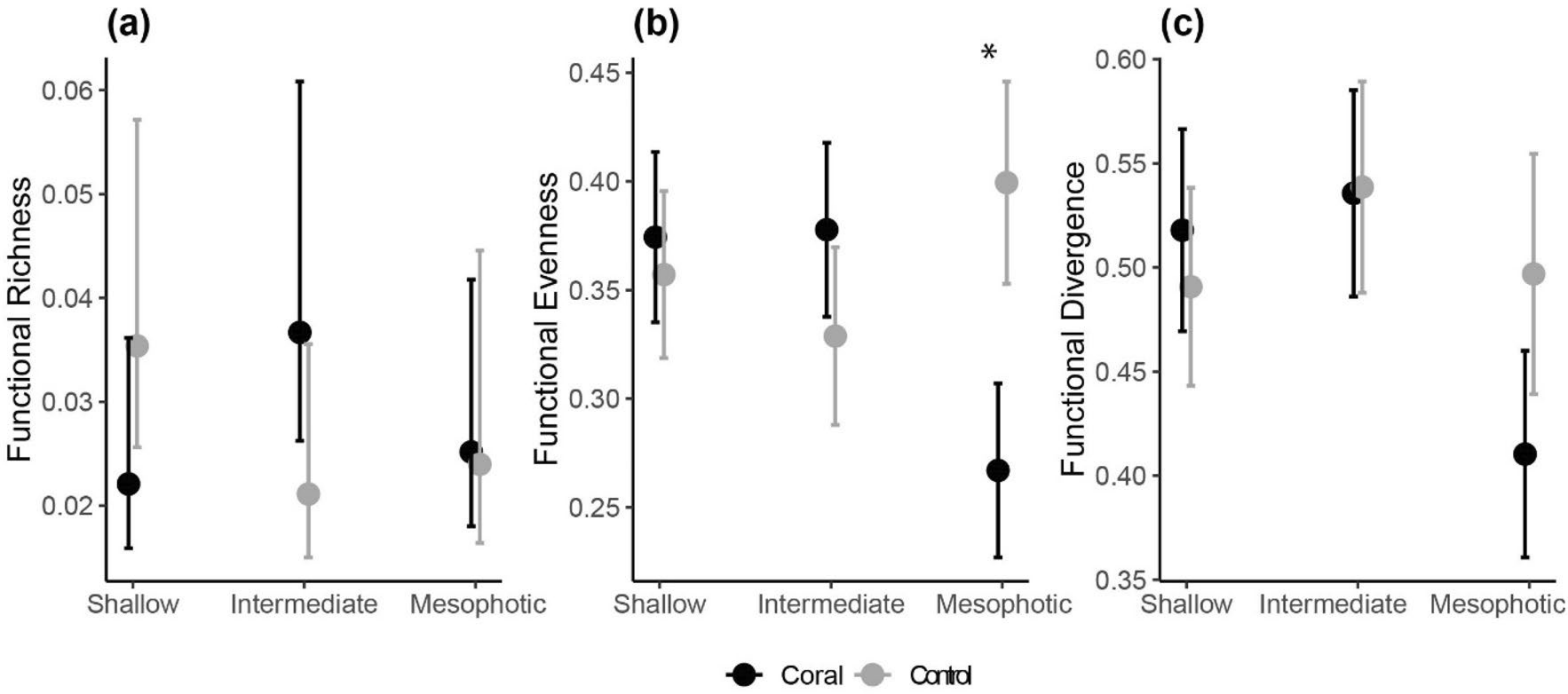
Predicted differences in functional (**a**) richness, (**b**) evenness, and (**c**) divergence among depth strata for coral (black) and control (grey) sites. Dots are model-averaged predictions from GLMMs, and whiskers are standard errors around the predicted mean. Significant differences (α = 0.05) between control and coral sites for each depth strata are indicated with an asterisk.

Despite filling a similar volume of the functional space (Fric, ‘depth x habitat,’ df = 2, *p* = 0.05, Table S6), shallow and intermediate reefs markedly differed from the mesophotic strata when accounting for the distribution of species abundances, a pattern that was driven by the presence of BCFs (Fig. 3). Species abundances in shallow and intermediate assemblages were more evenly distributed in the functional space. A higher proportion of this abundance was represented by species with extreme trait values (i.e. higher divergence) (Fig. 3b, c). In contrast, mesophotic fish assemblages within BCFs were characterized by higher dominance of species close to the centre of the trait space (i.e., lower evenness and divergence) (Fig. 3b, c). For FDiv, the presence of BCFs magnified differences between mesophotic reefs and their shallower counterparts, although there was high variability and overlapping confidence intervals (FDiv, ‘depth x habitat’, df = 2, *p* = 0.07, Table S6).

### Taxonomic and functional beta diversity

**Figure 4:**
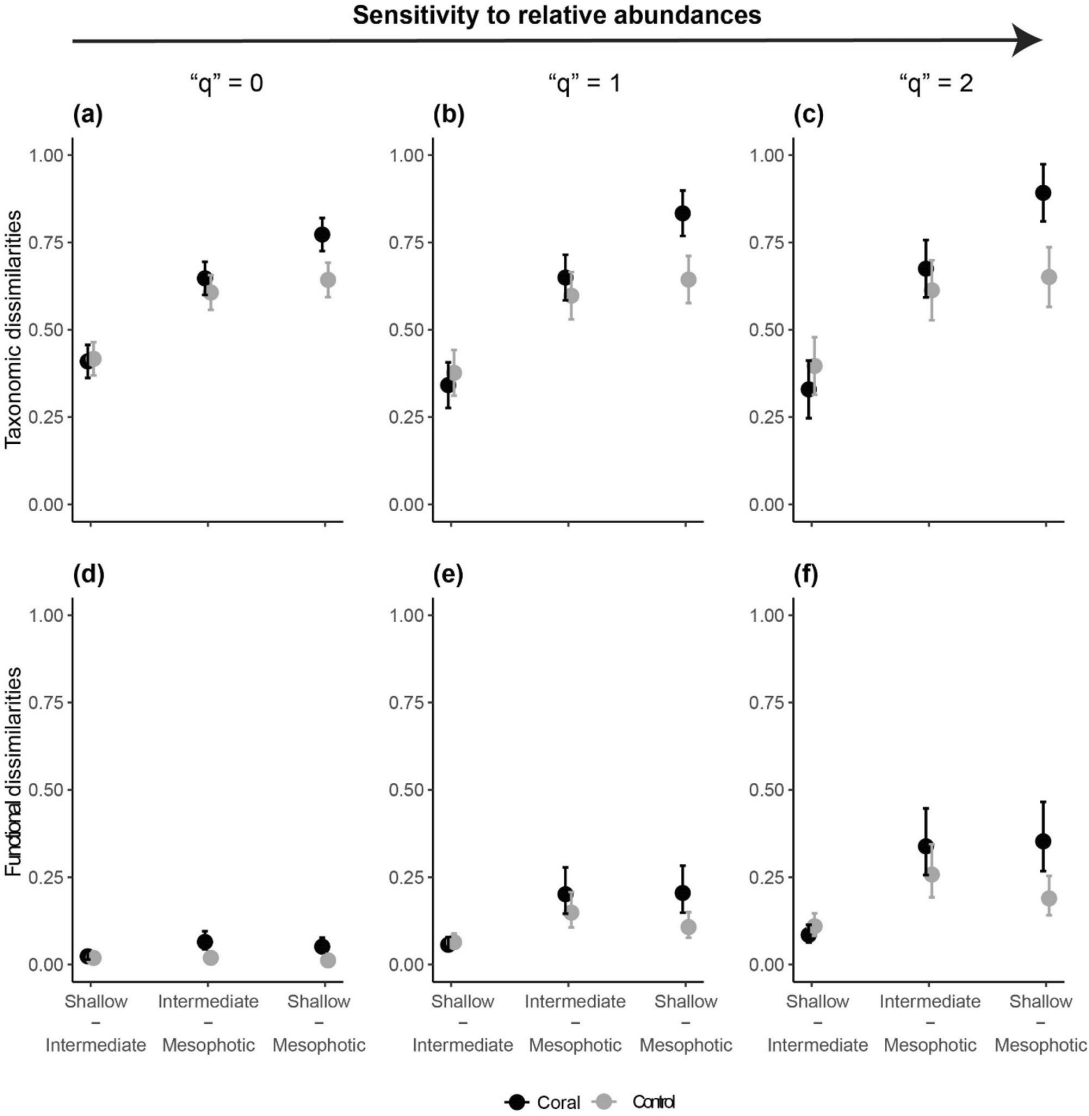
Predicted differences in the magnitude of (**a**–**c**) taxonomic and (**d**–**f**) functional dissimilarities among depth strata for coral (black) and control (grey) sites. Dissimilarities are shown under increasing sensitivity to taxonomic and functional entities relative abundances: (**a**, **d**) “q” = 0 (species composition only), (**b**, **e**) “q = 1” (higher weight on common species), and (**c**, **f**) “q” = 2 (higher weight on dominant species). Dots are model-averaged predictions from GLMMs, and whiskers are standard errors around the predicted mean.

The mesophotic strata hosted distinct compositions of species, a pattern that was enhanced by the presence of BCFs (‘depth × habitat’, df = 2, *p* < 0.01, Table S7). For compositional changes only (“q” = 0), the mesophotic strata shared, on average *ca.* 40% of the species with shallow and intermediate reefs (Fig. 4a). In contrast, most functional entities were shared between the mesophotic strata and their shallower counterparts (*ca.* 90% shared functional entities, Fig. 4d). Taxonomic and functional dissimilarities augmented when increasing the weight on relative abundances, suggesting changes in beta-diversity were driven mainly by shifts in the identity of common (“q” = 1) and dominant (“q” = 2) taxonomic and functional entities (Figs. 4b, c, e, f, S9). It is worth noting that taxonomic and functional dissimilarities between the mesophotic and shallow strata were magnified by the presence of BCFs (Fig. 4). For taxonomic dissimilarities, only *ca.* 20% common (“q” = 1) and 15% dominant (“q” = 2) species were shared with the shallow strata in BCFs. In contrast, mesophotic reefs where BCFs were absent shared *ca.* 40% of common and dominant species with shallow reefs. For functional dissimilarities, *ca.* 25% of common and *ca.* 35% of dominant functional entities were unique to mesophotic reefs containing BCFs. In contrast, mesophotic reefs where BCFs were absent only had *ca.* 10% and 20% of unique common and dominant functional entities respectively.

## Discussion

We provided one of the first empirical evidence of the community assembly processes involved in the generation and maintenance of the unique fish biodiversity associated with BCFs in subtropical oceanic islands. Few studies, to date, have documented trends in the diversity of fishes associated with mesophotic BCFs^35,37^, and, to the best of our knowledge, this is the first by means of a functional framework, providing insights on community assembly and potential flow-on effects on ecosystem functioning. This is a key aspect to understand the extent to which conservation of BCFs would enhance the breadth of functional roles supported by fishes over a regional context.

The taxonomic structure of fish assemblages shifted predictably across the depth gradient, with declines in species richness and shifts in species composition^10^. However, the degree of similarity in fish assemblage structure between the mesophotic and shallower strata depended on the presence of BCFs. Mesophotic fish assemblages inhabiting BCFs displayed limited overlap with their shallower counterparts, and lower multivariate dispersion. This indicates a fish assemblage that is more specialised and homogenous in terms of the identity and abundance of species. Like other marine animal forests^19^, BCFs can alter the physical and biogeochemical properties under their canopies through the complex three-dimensional architecture they provide, which can modify water flow, enhancing the retention of fish larvae and waterborne propagules that can serve as prey for adult fishes^20,38^. Thus, the structure provided by BCFs could stabilize the availability of resources across the seascape, making fish assemblages less variable across space. This stabilizing effect in the spatial distribution of resources is known to occur in other marine ‘ecosystem engineers’ such as saltmarshes^39^, kelp forests^40^, and seagrass meadows^41^.

The high taxonomic distinctiveness of fish assemblages within BCFs, compared to control sites, demonstrated the role of cold-water corals as an essential fish habitat for specialist species in mesophotic and deep-sea ecosystems^42,43^. This specialization is likely enhanced by the refuge and nursery effect provided by BCFs^19^. For instance, individuals of *Lapanella fasciata* were exclusively recorded swimming among black coral colonies, while *Anthias anthias* was almost two times more abundant above BCFs canopies (Fig. S9c, f), rapidly seeking refuge among the complex three-dimensional structure as the diver approached. This pattern seems to hold for BCFs at aphotic depths (*ca.* 300 m) in other Atlantic archipelagos, with these species having higher occurrence in BCFs^35,44^. Although the role of BCFs as important nurseries requires further scrutiny, we observed higher densities of small-sized individuals (< 5 cm) of the Hogfish (*Bodianus scrofa*) at the mesophotic strata within BCFs, compared to nearby areas with lower structural complexity (Fig. S10), as similarly reported for its congeneric *Bodianus insularis* in other Atlantic archipelagos^12^.

Despite hosting different species composition, mesophotic reefs filled a comparable volume of the trait space (i.e., functional richness), irrespective of the habitat, suggesting the maintenance of a similar range of trait combinations. This common ‘backbone’ appears to be a common feature of reef fish assemblages across latitudinal and depth gradients across disparate biogeographic regions^27,45,46^, suggesting the maintenance of a set of traits that underpin core ecosystem functions mediated by fishes^47^. Yet, when accounting for the distribution of species abundances, we found marked differences between the mesophotic strata and their shallower counterparts, a result that was driven by the presence of BCFs. Higher functional divergence and evenness in the shallower strata suggest a more complementary and efficient use of resources^36^, likely an outcome of lower abiotic constraints and higher energy availability (e.g., primary production) that enhances niche partitioning^25,26^. In contrast, higher abiotic (e.g., temperature, pressure) constraints and lower energy availability at the mesophotic strata may drive convergence towards optimal traits to maximize the use of available resources (e.g., relying heavily on planktonic supplies)^28,48^. It has also been hypothesized that functional convergence may be further enhanced by high dominance of taxa with ‘superior’ traits, that may exclude competitively ‘inferior’ species (i.e. “Weak Competitor Exclusion”^49^), a scenario that can be particularly important in areas with an homogenous distribution of resources^50^ such as BCFs. Our result showing that differences in the functional evenness and divergence of fish assemblages among depth strata are mediated by the presence of BCFs supports this prediction, and provides empirical evidence for the role of BCFs as key ‘ecosystem engineers’ in mesophotic reefs^19^.

Beyond these insights on community assembly, the lower evenness in the distribution of abundances in the trait space in mesophotic BCFs suggests that a larger breadth of potential ecosystem functions have limited insurance (i.e. lower redundancy) against selective human stressors than may cause local extinctions or drastic declines in population abundances^51^. This functional vulnerability is further enhanced by the limited overlap in species composition with shallow reefs^14,15^. Despite *ca.* 90% of functional entities were shared between the mesophotic strata and their shallower counterparts, there was limited number of shared species, particularly for common and very abundant species. This compositional shift suggests that, although core ecosystem functions of shallow reefs are represented in the mesophotic strata, most species would not be able to replenish these functions in shallow reefs, due to the environmental and biological constrains that limit their vertical distribution^52^. Further, the identity of common and dominant functional entities, also shifted across the depth gradient, magnified by the presence of BCFs. Given the disproportionate role that abundant species exerts in ecosystem functions via their traits^53^, together these results suggests that shallow and mesophotic reefs differ in the main ecosystem functions supported by fishes.

There are several caveats that limits the generality of our findings. First, our study was conducted over a small spatial scale, with no replication at the island (i.e., multiple locations within the island), archipelago (i.e., multiple islands within the archipelago), and basin scale (i.e., multiple oceanic islands across different ocean basins). Although this certainly limits the representativeness of the patterns reported here, similar patterns have been reported elsewhere for congeneric species that share ecological traits with the species reported here^13,35,44^. This highlights the value of species functional traits to understand ecological dynamics across biogeographic boundaries^24^, providing information that can be used to develop common management strategies for vulnerable BCFs ecosystems^54^. Second, the low detectability of cryptic species by UVCs might have underscored the importance of niche partitioning in mesophotic BCFs, as these species tend to partition resources very finely across available micro-niches^55^. Levelling-up survey efforts in MEs globally, using a combination of sampling methods that overcome the logistical problems of sampling at depths over large geographical scales (e.g., stereo-BRUVs^56^), and the bias associated with the detection of cryptic species (e.g., eDNA^57^), will provide further insights on the generality of the patterns reported here.

There is an urgent need to booster our understanding of the biodiversity and functioning of MEs^58^. Our study provides empirical evidence on the ecological mechanisms that have contributed to generate and maintain the unique biodiversity found on mesophotic MAFs. We have shown that mesophotic habitats dominated by BCFs promoted the specialization of reef fishes, likely linked to convergence towards optimal traits that enable them to maximize the use of resources and space. Given the vulnerability of these slow-growing marine animal forests to human impacts, such as destructive fishing (e.g., trawling), mining, coastal development, and sedimentation^19,23^, developing a portfolio of management and conservation strategies that specifically tackle their unique biodiversity and ecosystem functioning is required to maximize regional biodiversity.

## Methods

### Study area and design

The fieldwork took place off the southeastern coast of Lanzarote Island (the Canary Islands, eastern Atlantic Ocean), outside Puerto del Carmen (28°55′5′′ N, 13°40′24′′ W; Fig. 1a, b). This location is designated as a Special Area for Conservation under the Habitat directive of the EU Natura 2000 network, and was selected on the basis of previous records of the black coral *Antipathella wollastoni* (Gray, 1857) in the shallower limits of its distribution (*ca.* 50 m depth^33^). Local bottom topography is characterized by narrow rocky shelves and steep slopes, typical for oceanic volcanic islands^59^. Local hydrography is complex, with predominant tidal currents along a NW–SE direction (unpublished data), and northeast trade winds affecting shallow subtidal habitats by generating wind waves and near-bottom turbulence^60^. The high nutrient load transported by currents may affect the upper limit of black coral distribution, by either providing food or smothering the corals physically^61^.

Surveys were conducted in winter (February), spring (April) and autumn (October) 2021, during daylight hours (10 am to 4 pm), at three depth strata: shallow (0–10 m), intermediate (20–30 m), and mesophotic (60–70 m). These depth strata were selected based on previous studies on the distribution of *A. wollastoni* from Macaronesia^33^ and information for the study region on the diffuse attenuation coefficient (KdPAR) at optical depths Z1% and Z10% (Fig. S2). Data on KdPAR at optical depth Z1% was directly downloaded from Copernicus, at monthly intervals, from December 2020 to January 2023^62^. The coefficient Z10% was then calculated by applying the extinction coefficient obtained from the Z1% to the exponential relationship at 10% of light extinction. To test for the interactive effect of habitat and depth, we selected two sites where *A. wollastoni* was present (coral, herein) at the mesophotic strata and two sites where it was absent (control, herein) (Fig. 1b, c, d). At coral sites, black coral density and height (mean ± SE) ranged between 1.38 ± 0.24 to 1.71 ± 0.11 colonies·m^2^ and 114.52 ± 8.64 to 107.96 ± 6.15 cm respectively^33^. The percent cover of rocky and sandy bottoms was otherwise comparable between coral and control sites across depth zones (Fig. S1). Sites were separated by at least > 1 km to minimize non-independence of fish counts.

### Survey methods

Fish counts were conducted via underwater visual census techniques (UVCs), a non-destructive method carried out by scientific SCUBA divers with expertise in the regional ichthyofauna. All surveys were conducted by the same two divers (FO and FE) to minimize inter-observer variability, using closed-circuit rebreathers (Mini-Quamtum, Submatix, Germany) with gas mixes containing up to 60% helium for deeper dives. This method provides relatively comparable fish abundance and diversity estimates to traditional open-circuit systems, with the additional advantage to be better suited to capture mobile targeted species with diver avoidance behaviour^63^. At each site and depth strata, n = 4, 25 m long × 4 m wide (100 m^2^), belt transects were conducted following standard operating procedures for the study region^64^. Briefly, divers counted and identified, to the lowest possible taxonomic level (minimum genus level), all fish individuals within the transect area while swimming at a constant speed (Table S1), with fishes entering the field of view from behind the divers excluded to minimize double-counting. Sharks and rays (class Chondrichthyes) were excluded from the analyses due to their highly vagrant behavior and patchy distributions that challenge their population assessment via UVC^65^ (only representing 6% of the species initially recorded and 0.01% of the total abundance). Also, they have, markedly distinct life history, morphological, and ecological traits^66^ that might represent outliers in the multidimensional functional space. Due to the logistical challenges of sampling at depths, some transects were not conducted, resulting in a slightly unbalanced sampling design (Table S2). All survey methods were performed in accordance with relevant guidelines and regulations.

### Trait database

We compiled eleven functional traits for each fish species recorded from a recently published dataset on behavioural, morphological, and ecological characters of Atlantic reef fishes^66^. Traits selected represent key attributes of fish species that mediate their response to abiotic^27,67^, biotic^68^, and anthropogenic factors^51^, as well as their potential contribution to ecosystem functions^47^. These included: home range, diel activity, group size, level in the water column, preferred temperature, size class, body shape, caudal fin, mouth position, diet, and spawning strategy (Table S3). These traits were a priori selected based on their expected role in mediating species responses to changes in environmental, habitat, and anthropogenic factors across the depth gradient investigated (i.e. ‘Response framework’), as well as signaling potential shifts in core ecological processes that underpin the transfer of energy and materials (i.e. ‘Effect framework’)^69^ (details on trait levels and their expected role are provided in Table S3). Missing trait values were inferred from conspecific species, generally from the same geographic area, based on the published literature and the author’s combined knowledge of the species.

### Taxonomic structure

Non-metric multidimensional scaling (nm-MDS) was firstly used to visualise variation in fish assemblage structure (taxonomic-level) among depth strata for coral and control sites independently, using the ‘vegan’^70^ and ‘ggord’^71^ R packages. Ordination plots were based on a species-by-species Bray–Curtis dissimilarity matrix, with species abundances log10 (*x* + 1) transformed to balance the contribution of dominant and rare species. We used the first two dimensions with stress values converging after 64 (stress level = 0.13) and 43 (stress level = 0.16) iterations for coral and control assemblages, respectively. Ellipsoids were overlaid to depict confidence limits (0.95) areas encompassing depth strata. We then tested for variation in fish assemblage structure among depth strata as a function of habitat (two-way interaction, ‘depth x habitat’), using a model-based approach for multivariate abundance data^72^. This approach overcomes shortcomings related to mean–variance relationships, typical of distance-based approaches for multivariate community data (e.g., PERMANOVA and ANOSIM)^73^. Generalised linear models (GLMs), using a negative binomial distribution for overdispersed count data, were fitted to the species abundance matrix via the ‘mvabund’ R package^74^, with p-values calculated using 999 iterations via PIT-trap resampling. Violation of model assumptions was visually inspected by plotting residuals *vs.* fitted values.

### Functional structure

We built a multidimensional functional space based on the eleven traits to test for variation in the functional structure and diversity of fish assemblages. A principal coordinate analysis (PCoA) was computed on a species-by-species Gower distance matrix, which is able to accommodate ordinal, nominal, and quantitative traits. We chose the first four PCoA axes as the optimal number of dimensions, as they were the most parsimonious choice that minimized the mean absolute deviation (MAD = 0.045, Fig. S4) between the original trait-based distances and the Euclidean distances in the functional space^75^. The correlation between individual traits and PCoA axes was calculated using a Kruskal–Wallis test for categorical traits, and an r^2^ statistic from simple linear regressions for continuous traits.

### Functional alpha diversity

We tested for variation in within (alpha) fish diversity, by computing three complementary aspects of functional diversity, based on the multidimensional functional space computed for each transect: richness (Fric), evenness (Feve), and divergence (Fdiv)^36^. Functional richness represents the proportion of the functional space occupied by each assemblage and was calculated as the volume inside a convex-hull connecting all the species present in that assemblage. Functional evenness represents the regularity in the distribution of abundance in the multidimensional functional space and was calculated as the minimum spanning tree linking all the species present in that assemblage. Functional divergence represents the proportion of abundance supported by species with extreme trait values (i.e., those in the edges of the convex hull). These three dimensions allowed us to investigate both compositional (presence/absence) and structural (abundance) changes in the functional diversity of reef fish assemblages, providing insights in community assembly and ecosystem functioning^36^. Differences in functional alpha diversity among depth strata, as a function of habitat (two-way interaction, ‘depth x habitat’), were then tested via Generalised Linear Mixed Effects Models (GLMMs), implemented in the ‘glmmTMB’ R package^76^. Before analyses, we applied an exponential transformation to Feve and Fdiv, as values were highly left-skewed. Models were run using a “Gamma” error distribution with an “inverse” link function for Fric, while Feve and FDiv were fitted using a “Gaussian” distribution. All models included “Site” nested within each habitat, as well as “Season”, as random effects to control for spatial and temporal non-independence. Violation of model assumptions was visually inspected through plots of residuals *vs.* fitted values. Analyses of trait spaces and functional diversity were carried in the ‘mFD’ R package^77^.

### Taxonomic and functional beta diversity

We investigated whether fish assemblages associated with BCFs were taxonomically and functionally more distinctive, relative to their shallower counterparts, using an attribute diversity framework^78^. This framework, based on generalizations of Hill numbers, enables comparison of taxonomic (i.e., species) and functional (i.e., species within a pre-defined threshold of functional distinctiveness) entities shared among depth strata as a function of habitat. Here, we set the threshold value for defining functional entities as the averaged trait dissimilarities between species in the Gower distance matrix^79^. Beta diversity was calculated as normalized Sørensen dissimilarities, which range from 1, when two assemblages do not share any taxonomic and/or functional entities to 0, when all entities are shared. To decouple the importance of compositional (presence/absence) vs*.* structural (abundance) changes in driving patterns of dissimilarities, beta diversity was calculated as a function of a parameter “q”, which controls the sensitivity of the metrics to species’ and functional entities relative abundances^79^. When ‘q’ = 0, dissimilarities are insensitive to changes in abundance structure (i.e., analogous to an occurrence-based approach), while increasing “q” (i.e., “q” > 0) places more weight on the distribution of abundances among common (“q” = 1) and dominant (“q” = 2) entities. Differences in the magnitude of taxonomic and functional dissimilarities among depth strata as a function of habitat (two-way interaction, ‘depth × habitat’) were then tested via GLMMs, implemented in the ‘glmmTMB’ R package^76^, for each metric and “q” parameter independently. Models were run using a “tweedie” error distribution, which is adequate for handling the high proportion of assemblages with low values of dissimilarities and overdispersion in the data, and included the same random effect structure as models for functional alpha diversity. Violation of model assumptions was visually inspected through plots of residuals *vs.* fitted values. Indices of beta diversity were calculated using functions in the ‘mFD’ R package^77^.

## Supplementary Information

Supplementary Information.

## Data Availability

All data and R code used for analyses will be made available through the author’s personal GitHub repository (https://github.com/NestorBosch) after acceptance of the manuscript.
