# Supplementary material for: Black coral forests enhance taxonomic and functional distinctiveness of mesophotic fishes in an oceanic island: implications for biodiversity conservation
Source: Sci Rep. 2023 Mar 27;13:4963. doi: 10.1038/s41598-023-32138-x (PMC10043018; doi:10.1038/s41598-023-32138-x)
Supplement: Supplementary file 1 — Supplementary Information. [file 41598_2023_32138_MOESM1_ESM.docx]

**Black coral forests enhance taxonomic and functional distinctiveness of mesophotic fishes in an oceanic island: implications for biodiversity conservation**


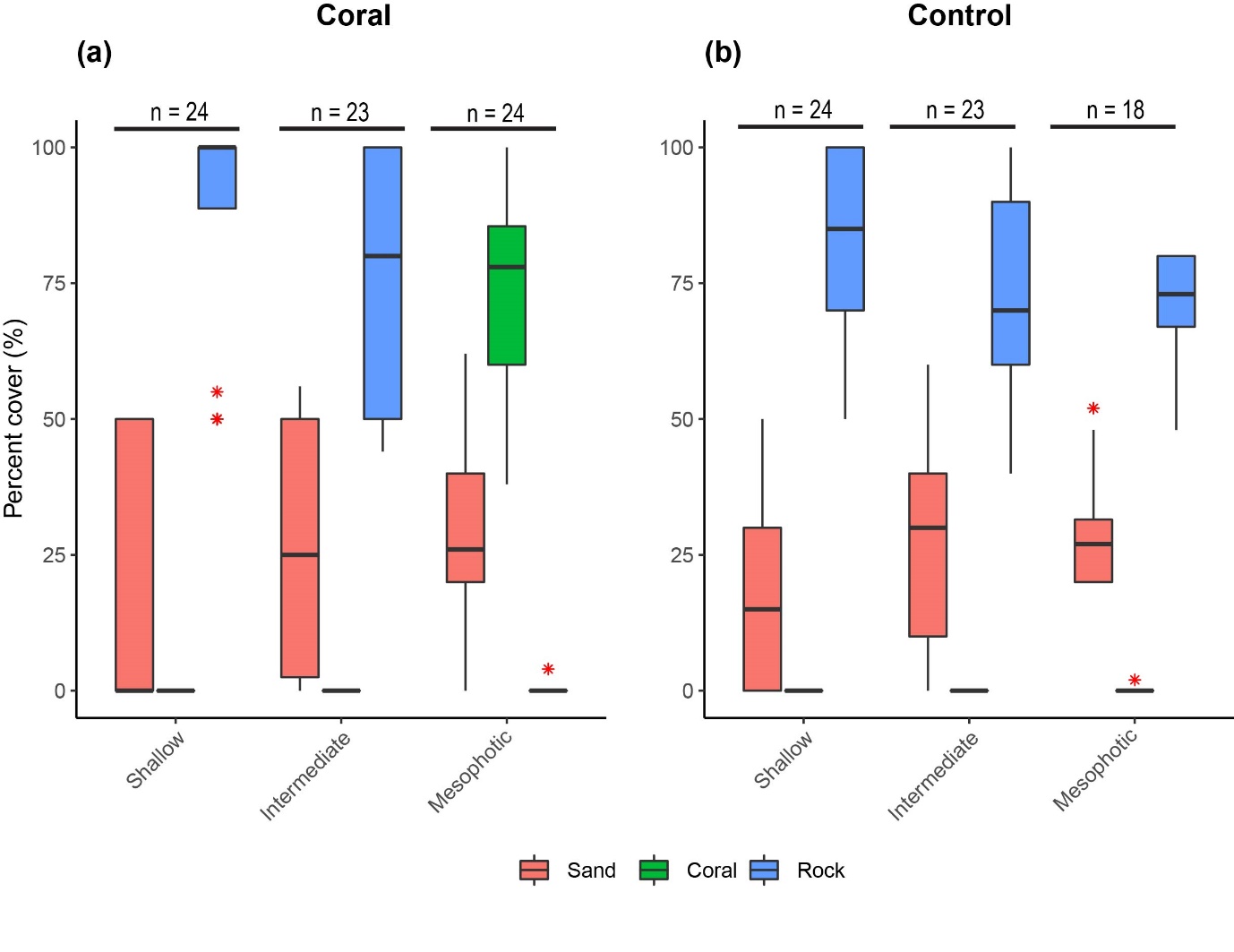


**Figure S1.** Boxplot distributions of percent cover (%) of habitat categories across depth strata. Red asterisks indicates outliers.


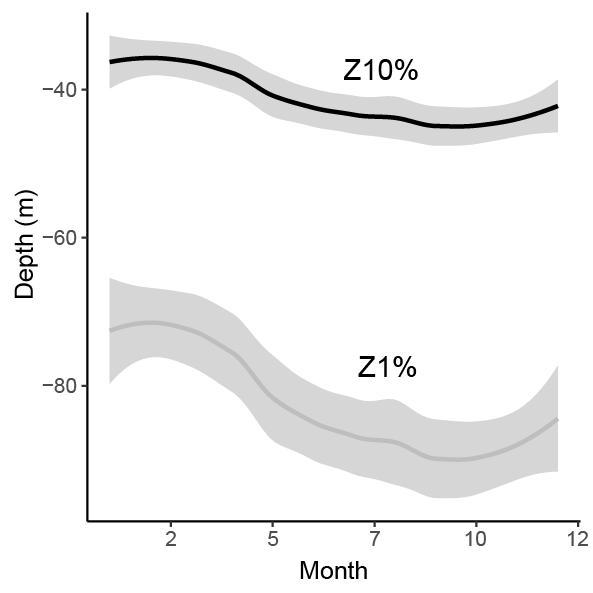


**Figure S2.** Intra-annual variation of optical depths Z10% (black line) and Z1% (gray line) based on data on the diffuse attenuation coefficient (KdPAR) downloaded from Copernicus for the study region (SE coast of Lanzarote Island).


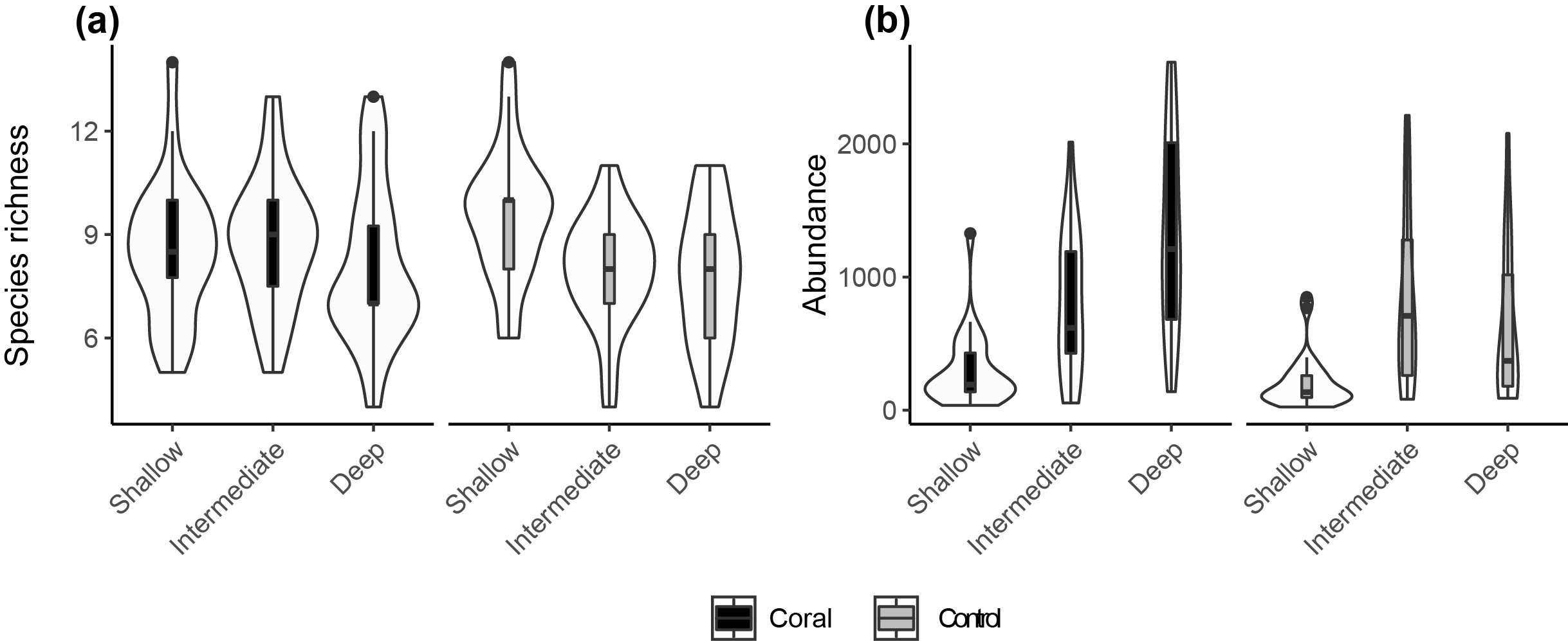


**Figure S3.** Variation on fish (a) species richness and (b) abundance across depth strata for coral (black) and control (gray) sites. Violin plots and boxplots are shown to depict the distribution of richness and abundance values at each depth strata for coral and control sites.


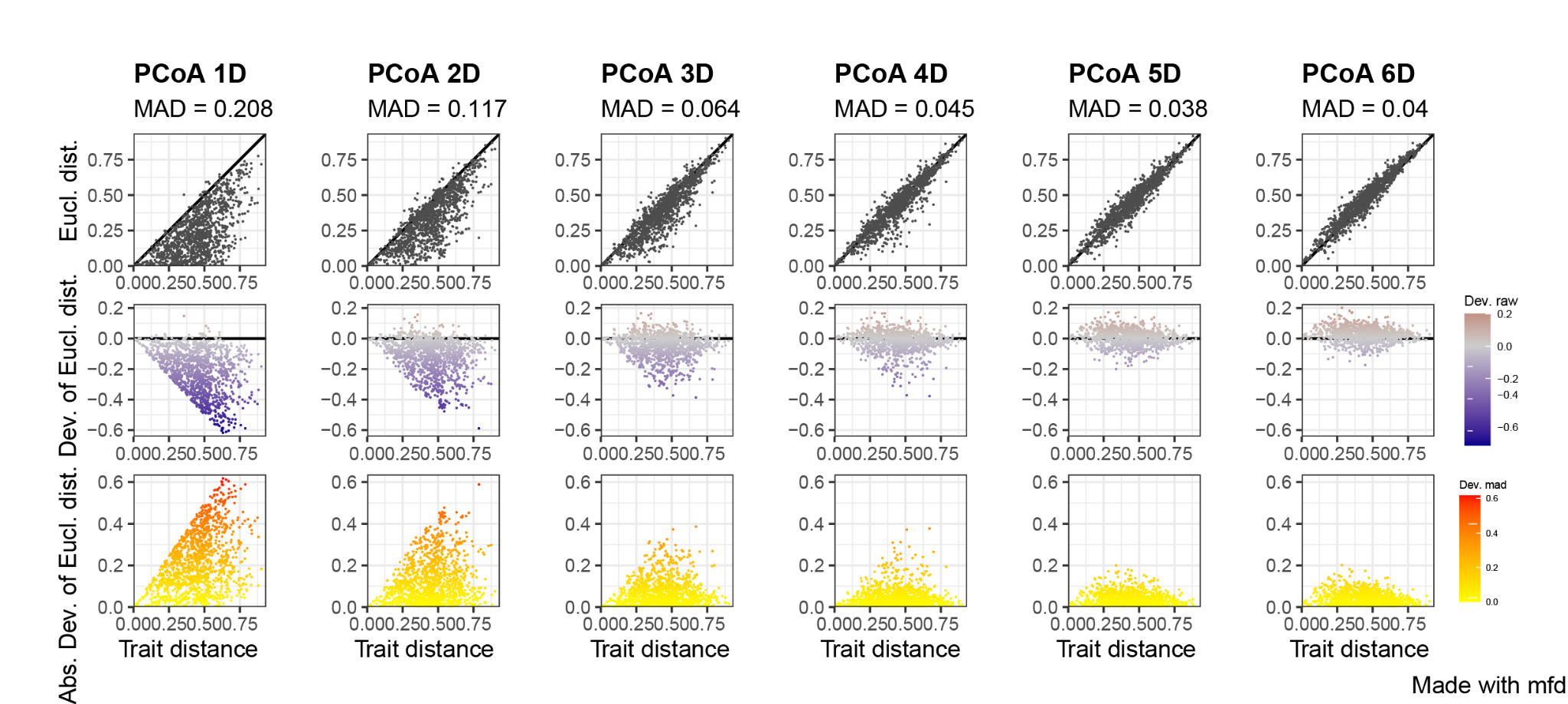


**Figure S4.** Scatterplots showing the quality of the multidimensional trait space with increasing number of PCoA axes. The first row depicts species functional distances in the multidimensional space. The second row depicts the raw deviation of species distances in the functional space compared to trait-based distances. The third raw depicts the absolute deviation of the distance in the functional space. The mean absolute deviations (MADs) between the original trait-based distances and the Euclidean distances in the functional space are indicated at the top for each number of PCoA dimensions.


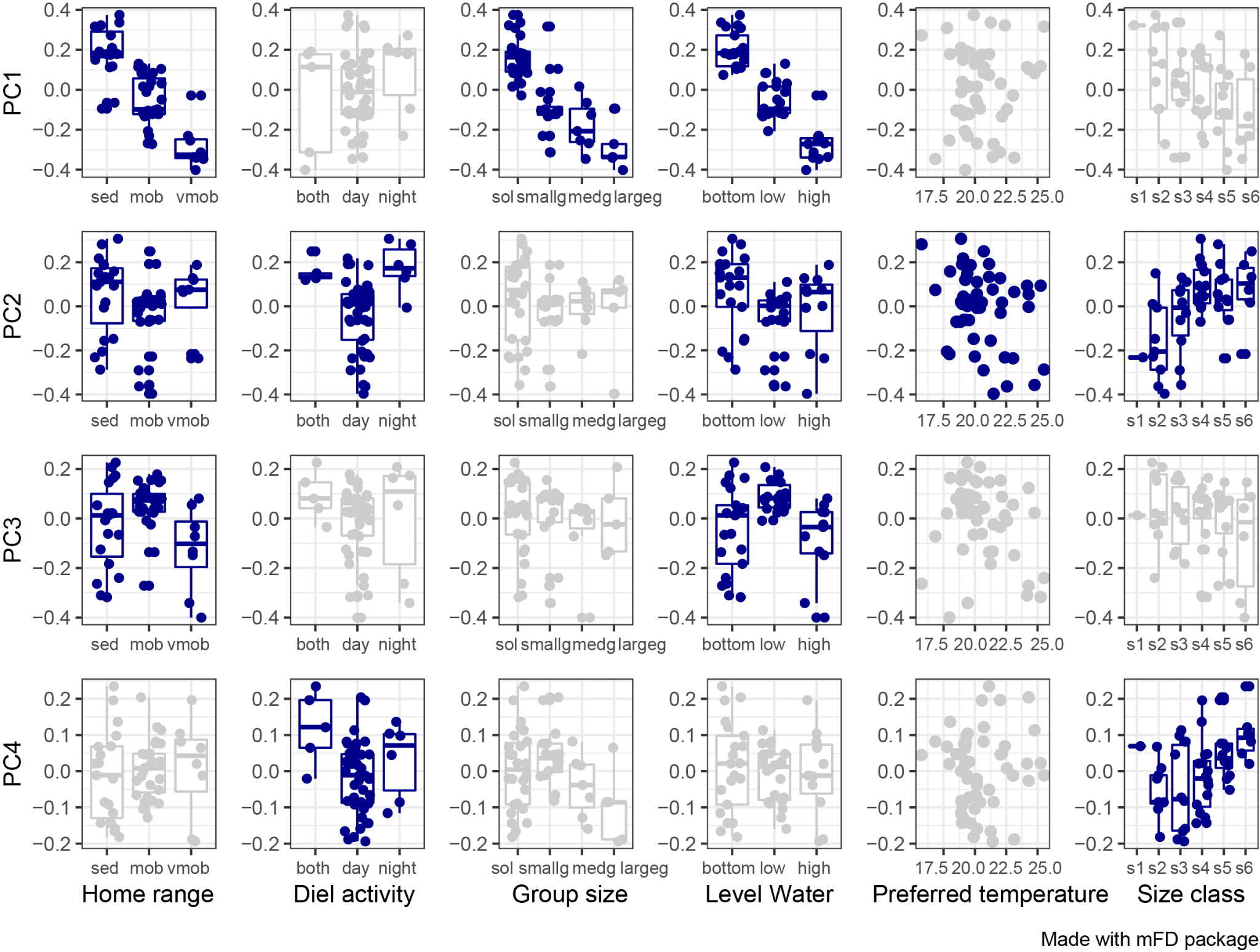


**Figure S5.** Boxplot illustrating the direction of the correlation (Table S4) between individual traits and PCoA axes. Significant trait-PCoA axes correlations are plotted in blue, while non-significant correlations are plotted in grey.


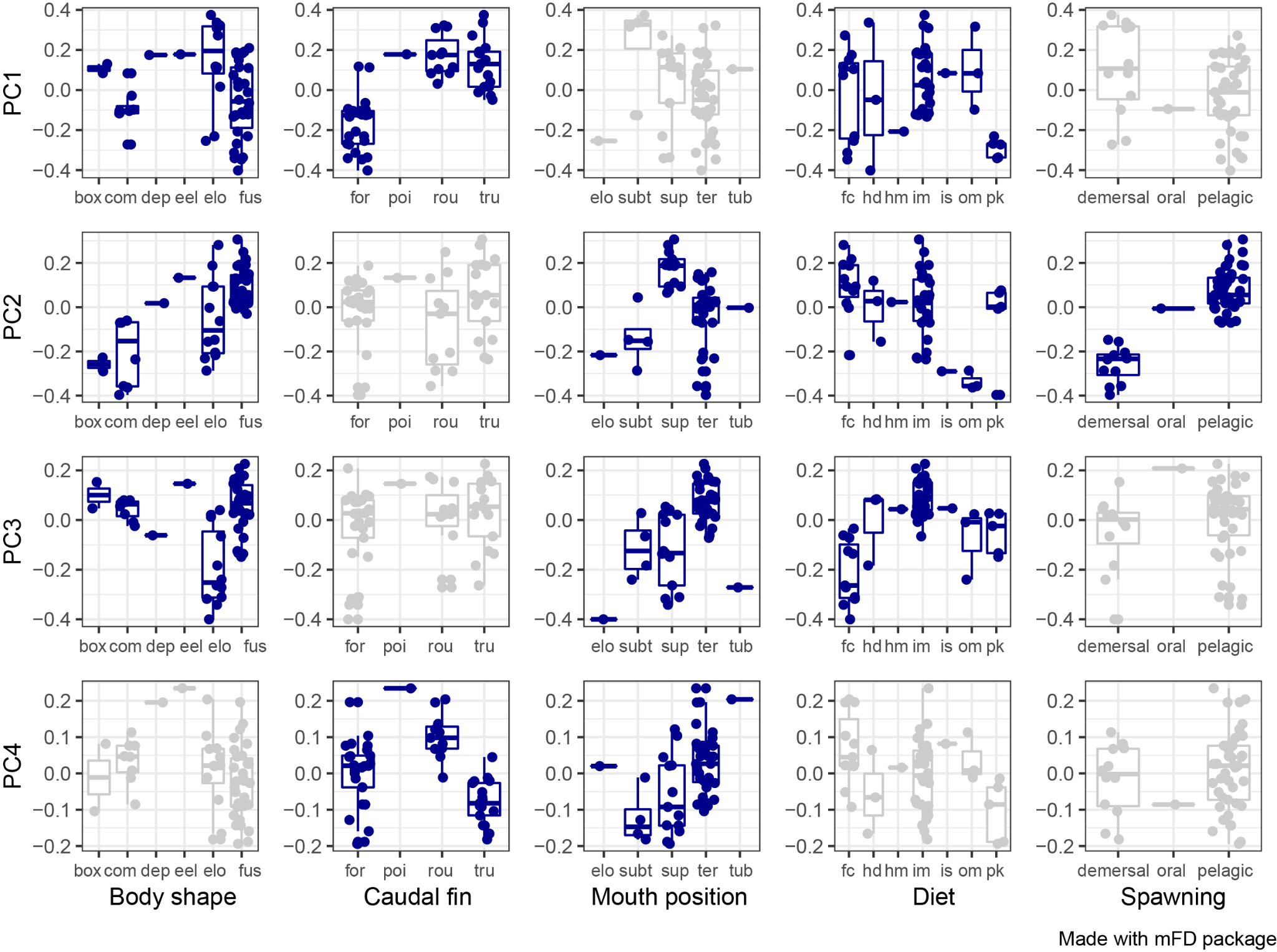


**Figure S6.** Boxplot illustrating the direction of the correlation (Table S4) between individual traits and PCoA axes. Significant trait-PCoA axes correlations are plotted in blue, while non-significant correlations are plotted in grey.


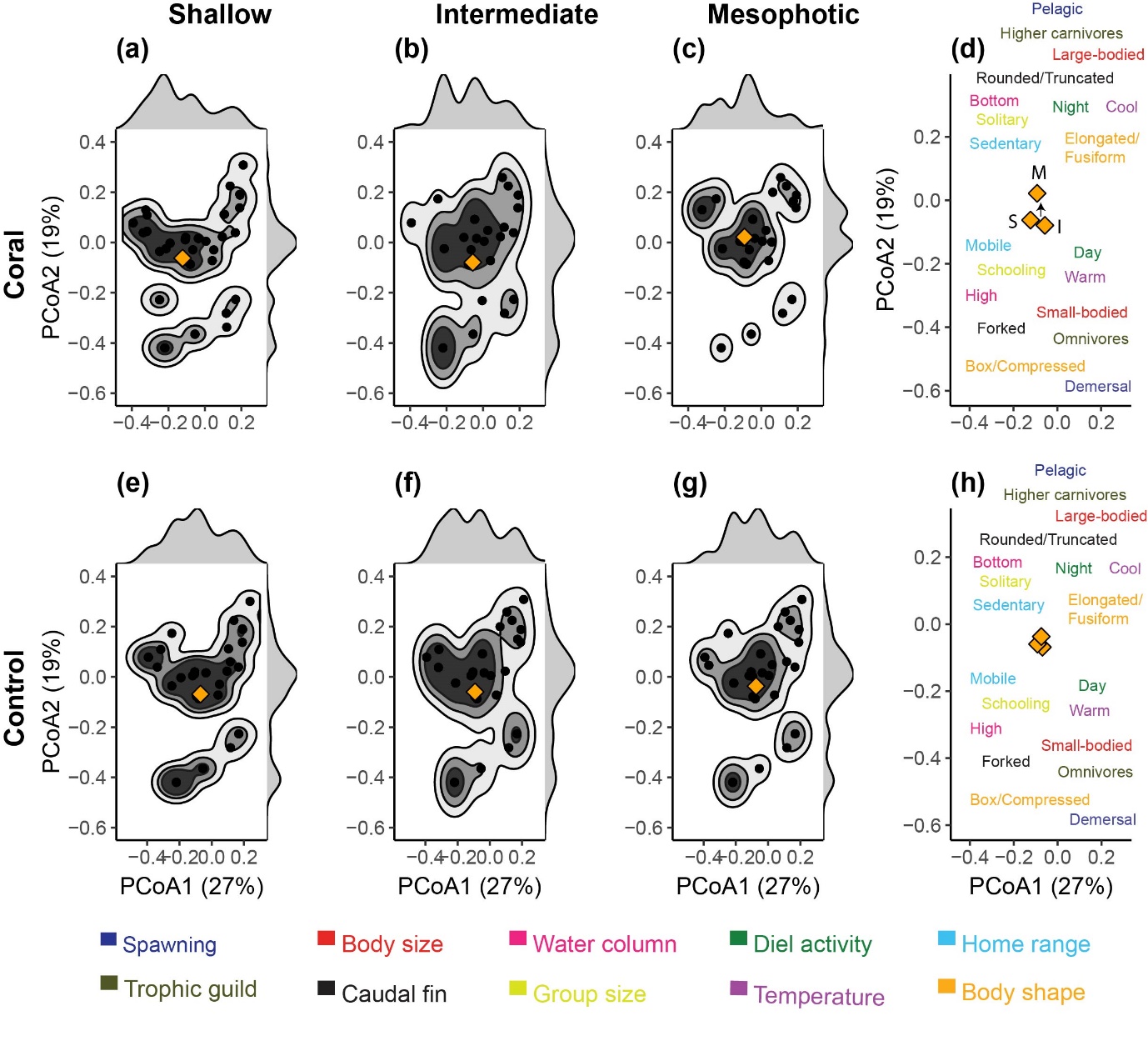


**Figure S7.** Trait space showing the position of fish species recorded at (a, e) shallow, (b, f) intermediate, and (c, g) mesophotic strata along the first and second PCoA axes for (a - d) coral and (e – h) control sites. Contours depict the 50% (dark), 75% (grey), and 95% (light grey) highest density intervals (HDIs) on each PCoA distribution. Marginal distributions for PCoA1 and PCoA2 are provided in each panel. Orange diamonds illustrate the abundance-weighted position of shallow (S), intermediate (I), and mesophotic (M) fish assemblages in the trait space. In (d, h), the centroid movement is represented in the trait space (arrows), with the most relevant levels for each trait mediating this shift (Table S4, S5) indicated with different colours.

**
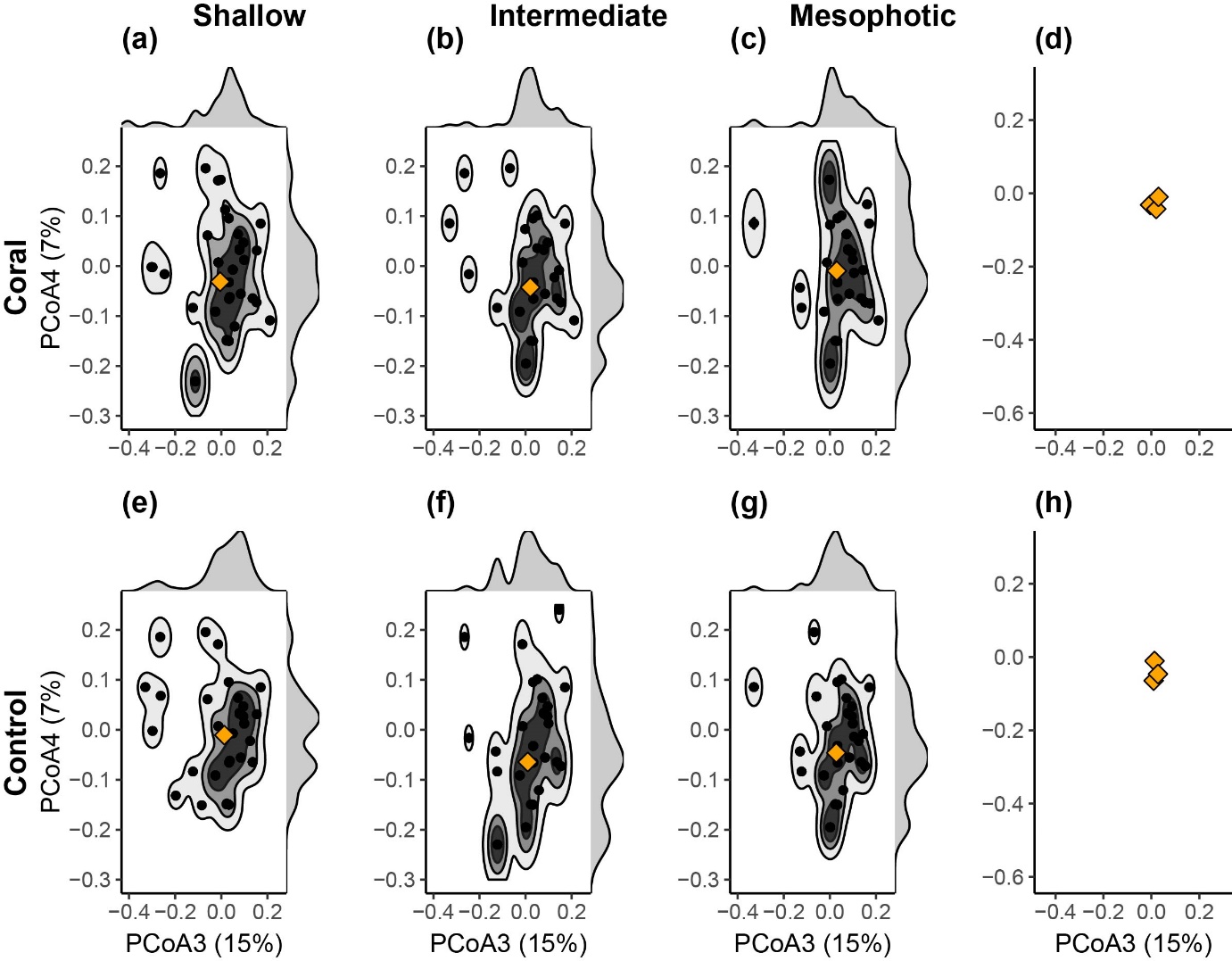
**

**Figure S8.** Trait space showing the position of fish species recorded at (a, e) shallow, (b, f) intermediate, and (c, g) mesophotic strata along the third and fourth PCoA axes for (a - d) coral and (e – h) control sites. Contours depict the 50% (dark), 75% (grey), and 95% (light grey) highest density intervals (HDIs) on each PCoA distribution. Marginal distributions for PCoA3 and PCoA4 are provided in each panel. Orange diamonds illustrate the abundance-weighted position of each assemblage in the trait space. In (d, h), the centroid movement is represented in the trait space (arrows).

**
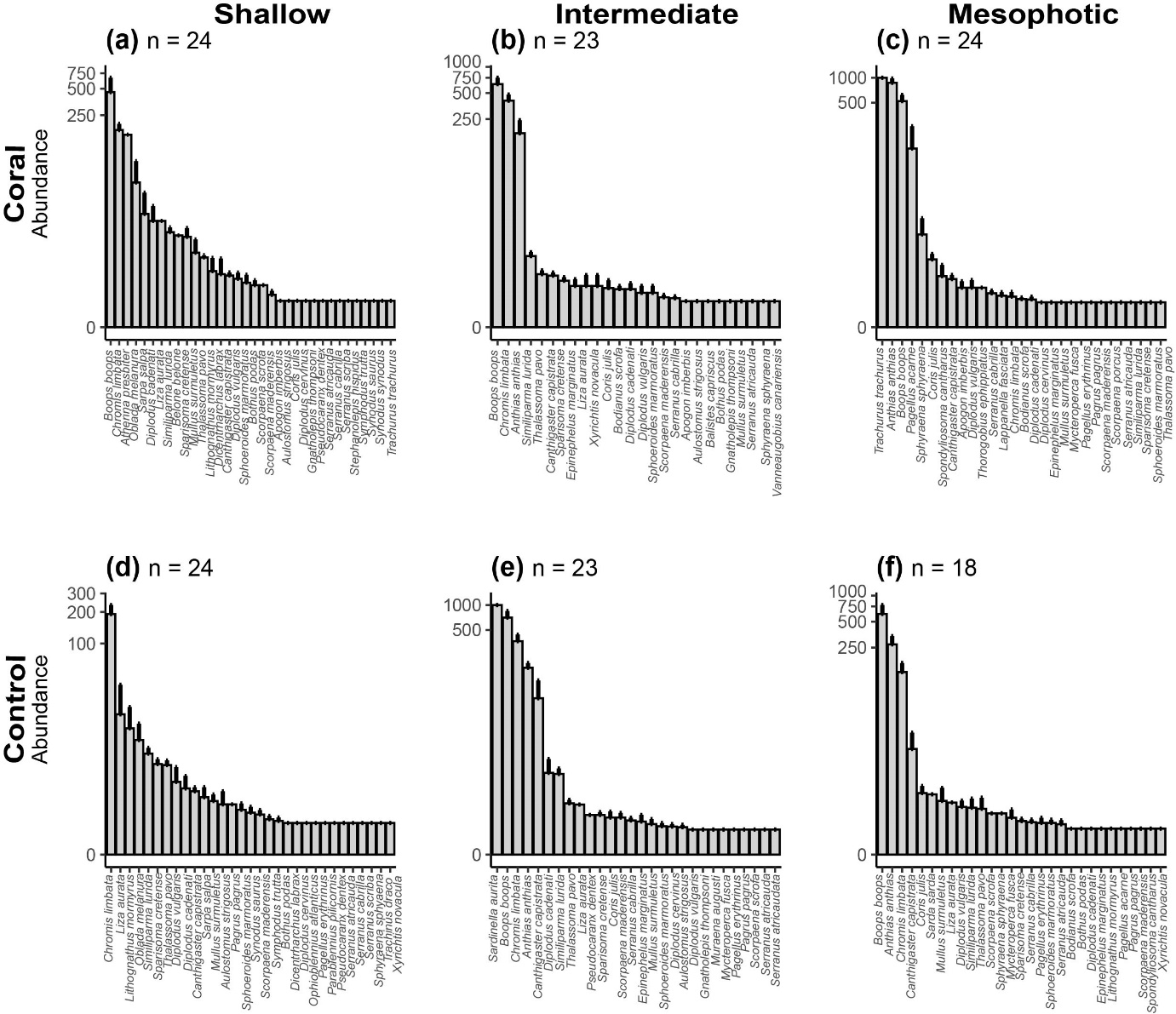
**

**Figure S9.** Mean (+ SE) fish species abundances (ind. × 100m^-2^) at the (a, d) shallow, (b, e) intermediate, and (c, f) mesophotic strata for (a – c) coral and (d – f) control sites. For visualization purposes, the y-axis is shown in log-scale.


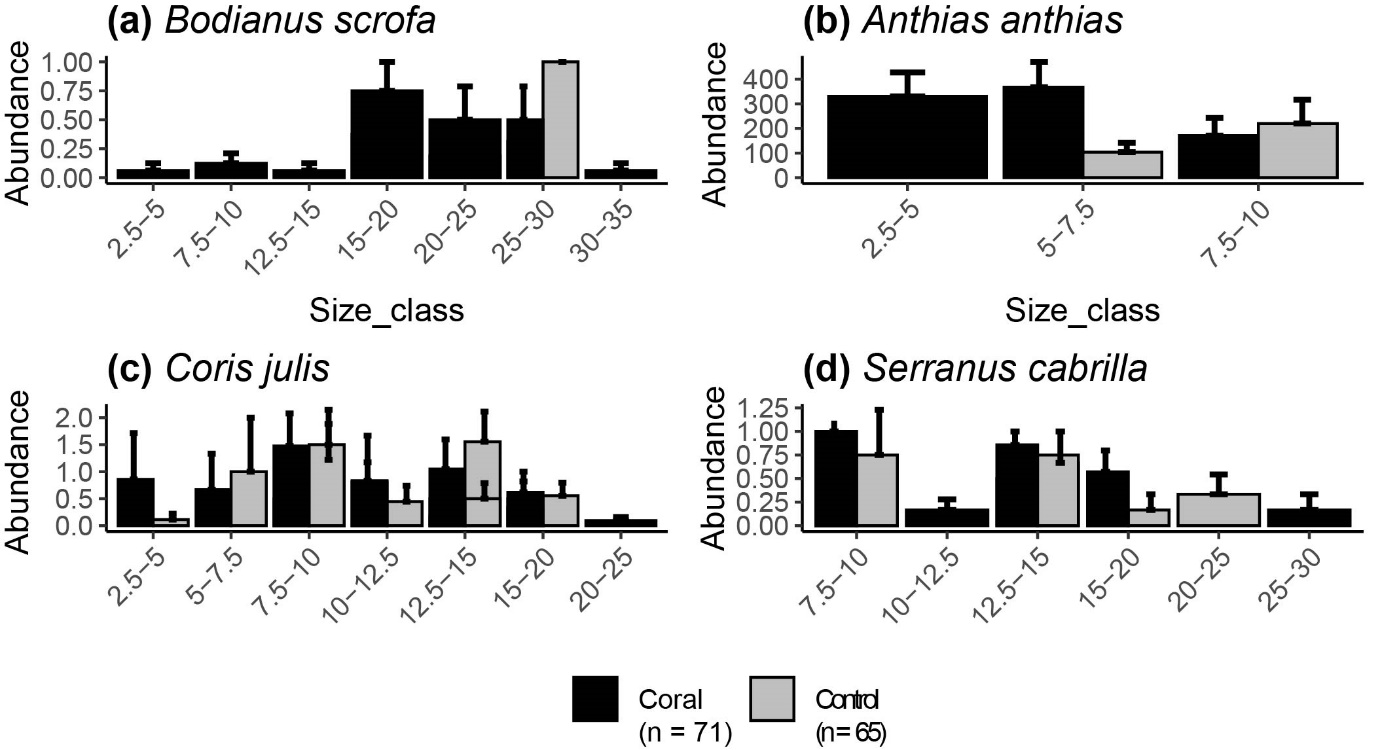


**Figure S10.** Mean (+ SE) abundances (ind. × 100m^-2^) of (a) *Bodianus scrofa*, (b) *Anthias anthias*, (c) *Coris julis*, and (d) *Serranus cabrilla* at the mesophotic strata at coral (black) and control (grey) sites.

**Table S1.** List of fish species recorded and their allocated trait values. Abbreviation of trait levels are provided in table S3.

| **Species** | **Home range** | **Diel activity** | **Group size** | **Water Column** | **Preferred Temperature** | **Size class** | **Body shape** | **Caudal fin** | **Mouth position** | **Diet** | **Spawning** |
| --- | --- | --- | --- | --- | --- | --- | --- | --- | --- | --- | --- |
| *Atherina presbiter* | vmob | day | largeg | high | 21.42 | s3 | fusiform | forked | superior | pk | pelagic |
| *Anthias anthias* | sed | day | medg | low | 20.17 | s3 | fusiform | forked | superior | im | pelagic |
| *Apogon imberbis* | sed | night | largeg | low | 20.41 | s2 | fusiform | forked | terminal | im | oral |
| *Aulostomus strigosus* | mob | day | smallg | bottom | 24.29 | s5 | elongated | rounded | tubular | fc | pelagic |
| *Balistes capriscus* | vmob | day | sol | high | 23.05 | s5 | compressed | truncated | terminal | im | demersal |
| *Belone belone* | vmob | day | medg | high | 17.96 | s6 | elongated | forked | elongated | fc | demersal |
| *Bodianus scrofa* | mob | day | sol | low | 20.16 | s5 | fusiform | truncated | terminal | im | pelagic |
| *Boops boops* | mob | day | smallg | high | 19.4 | s4 | fusiform | forked | terminal | pk | pelagic |
| *Bothus podas* | sed | day | sol | bottom | 20.55 | s4 | depressed | rounded | terminal | fc | pelagic |
| *Canthigaster capistrata* | mob | day | sol | low | 19.99 | s2 | box_shaped | truncated | terminal | im | demersal |
| *Chromis limbata* | mob | day | largeg | high | 21.49 | s2 | compressed | forked | terminal | pk | demersal |
| *Coris julis* | mob | day | smallg | low | 18.91 | s3 | fusiform | truncated | terminal | im | pelagic |
| *Dicentrharchus labrax* | vmob | day | medg | high | 16.84 | s6 | fusiform | forked | terminal | fc | pelagic |
| *Diplodus cadenati* | mob | day | smallg | low | 18.52 | s4 | compressed | forked | terminal | im | pelagic |
| *Diplodus cervinus* | mob | day | smallg | low | 19.14 | s5 | compressed | forked | terminal | im | pelagic |
| *Diplodus vulgaris* | mob | day | smallg | low | 18.85 | s4 | compressed | forked | terminal | im | pelagic |
| *Epinephelus marginatus* | mob | both | sol | bottom | 20.71 | s6 | fusiform | rounded | superior | im | pelagic |
| *Gnatholepis thompsoni* | sed | day | smallg | bottom | 25.52 | s2 | elongated | rounded | subterminal | om | demersal |
| *Lappanella fasciata* | mob | day | sol | low | 18.99 | s2 | fusiform | truncated | terminal | im | pelagic |
| *Liza aurata* | vmob | both | largeg | high | 19.23 | s5 | fusiform | forked | terminal | hd | pelagic |
| *Lithognathus mormyrus* | mob | day | smallg | low | 19.55 | s5 | fusiform | forked | terminal | im | pelagic |
| *Mullus surmuletus* | mob | day | medg | low | 19.07 | s4 | fusiform | forked | subterminal | im | pelagic |
| *Muraena augusti* | sed | both | sol | bottom | 21.13 | s6 | eel_like | pointed | terminal | im | pelagic |
| *Mycteroperca fusca* | mob | day | sol | bottom | 21.13 | s5 | fusiform | truncated | superior | fc | pelagic |
| *Oblada melanura* | mob | day | medg | high | 20.16 | s4 | fusiform | forked | terminal | pk | pelagic |
| *Ophioblennius atlanticus* | sed | day | sol | bottom | 21.26 | s3 | elongated | truncated | subterminal | hd | demersal |
| *Pagellus acarne* | mob | day | smallg | low | 18.89 | s4 | fusiform | forked | terminal | im | pelagic |
| *Pagellus erythrinus* | mob | day | smallg | low | 19.27 | s5 | fusiform | forked | terminal | im | pelagic |

| **Species** | **Home range** | **Diel activity** | **Group size** | **Water Column** | **Preferred Temperature** | **Size class** | **Body shape** | **Caudal fin** | **Mouth position** | **Diet** | **Spawning** |
| --- | --- | --- | --- | --- | --- | --- | --- | --- | --- | --- | --- |
| *Pagrus pagrus* | mob | day | smallg | low | 22.16 | s6 | fusiform | forked | terminal | im | pelagic |
| *Parablennious pilicornis* | sed | day | sol | bottom | 19.9 | s2 | elongated | truncated | subterminal | im | demersal |
| *Pseudocaranx dentex* | vmob | both | smallg | high | 22.01 | s5 | fusiform | forked | terminal | fc | pelagic |
| *Sarda sarda* | vmob | day | medg | high | 10.7 | s6 | fusiform | forked | terminal | fc | pelagic |
| *Sardinella aurita* | vmob | day | largeg | high | 23.14 | s3 | fusiform | forked | superior | pk | pelagic |
| *Sarpa salpa* | mob | day | medg | low | 19.2 | s5 | fusiform | forked | terminal | hm | pelagic |
| *Scorpaena canariensis* | sed | both | sol | bottom | 19.39 | s2 | fusiform | truncated | terminal | im | pelagic |
| *Scorpaena maderensis* | sed | night | sol | bottom | 19.33 | s3 | fusiform | rounded | terminal | im | pelagic |
| *Scorpaena porcus* | sed | night | sol | bottom | 18.02 | s4 | fusiform | rounded | terminal | im | pelagic |
| *Scorpaena scrofa* | sed | night | sol | bottom | 18.89 | s4 | fusiform | truncated | superior | im | pelagic |
| *Serranus atricauda* | sed | day | sol | bottom | 19.66 | s4 | fusiform | truncated | superior | im | pelagic |
| *Serranus cabrilla* | sed | day | sol | bottom | 19.05 | s4 | fusiform | truncated | superior | fc | pelagic |
| *Serranus scriba* | sed | day | sol | bottom | 18.98 | s4 | fusiform | truncated | superior | im | pelagic |
| *Similiparma lurida* | mob | day | smallg | low | 22.56 | s2 | compressed | forked | terminal | om | demersal |
| *Sparisoma cretense* | mob | day | smallg | low | 20.07 | s4 | fusiform | truncated | terminal | hd | pelagic |
| *Sphoeroides marmoratus* | mob | day | sol | low | 20.69 | s3 | box_shaped | rounded | terminal | is | demersal |
| *Sphyraena sphyraena* | vmob | night | smallg | high | 19.42 | s6 | elongated | forked | superior | fc | pelagic |
| *Spondyliosoma cantharus* | mob | day | smallg | low | 19.29 | s5 | compressed | forked | terminal | im | pelagic |
| *Stephanolepis hispidus* | mob | day | sol | low | 24.76 | s3 | compressed | rounded | terminal | om | demersal |
| *Symphodus trutta* | mob | day | sol | low | 22.05 | s3 | fusiform | rounded | terminal | im | pelagic |
| *Synodus saurus* | sed | day | sol | bottom | 24.16 | s4 | elongated | forked | superior | fc | pelagic |
| *Synodus synodus* | sed | day | sol | bottom | 25.25 | s4 | elongated | forked | superior | fc | pelagic |
| *Thalassoma pavo* | mob | day | medg | low | 19.53 | s3 | elongated | truncated | terminal | im | pelagic |
| *Thorogobius ephippiatus* | sed | day | sol | bottom | 17.77 | s2 | elongated | rounded | terminal | im | demersal |
| *Trachinus draco* | sed | night | sol | bottom | 15.74 | s5 | elongated | truncated | superior | fc | pelagic |
| *Trachurus trachurus* | vmob | both | smallg | high | 17.34 | s5 | fusiform | forked | terminal | fc | pelagic |
| *Vanneaugobious canariensis* | sed | day | sol | bottom | 22.39 | s1 | elongated | rounded | terminal | im | demersal |
| *Xyrichtis novacula* | mob | day | sol | bottom | 24.29 | s4 | fusiform | truncated | terminal | im | pelagic |

**Table S2.** Distribution of sampling effort (i.e., number of UVCs transects) per depth strata across seasons for coral and control sites.

|  | **Coral** | | | | | | **Control** | | | | | |
| --- | --- | --- | --- | --- | --- | --- | --- | --- | --- | --- | --- | --- |
|  | **Winter** | | **Spring** | | **Autumn** | | **Winter** | | **Spring** | | **Autumn** | |
|  | **Site 1** | **Site 2** | **Site 1** | **Site 2** | **Site 1** | **Site 2** | **Site 3** | **Site 4** | **Site 3** | **Site 4** | **Site 3** | **Site 4** |
| **Shallow** | 4 | 4 | 4 | 4 | 4 | 4 | 4 | 4 | 4 | 4 | 4 | 4 |
| **Intermediate** | 4 | 4 | 4 | 4 | 4 | 3 | 4 | 4 | 4 | 4 | 3 | 4 |
| **Mesophotic** | 4 | 4 | 4 | 4 | 4 | 4 | 2 | 4 | 2 | 4 | 2 | 4 |

**Table S3.** List of reef fish functional traits used, including their role in mediating species responses to environmental, biotic, and/or anthropogenic factors (‘Response framework’), and contributions to ecosystem functions (‘Effect framework’).

| **Type** | **Trait** | **Category** | **Levels** | **Response framework** | **Effect framework** |
| --- | --- | --- | --- | --- | --- |
| Behavioural | Home range | Ordinal | Sedentary (sed), mobile (mob), very mobile (vmob) | Mediates the ability of fishes to cope with periods of environmental stress (e.g. tracking shifts in climate) (1), and their vulnerability to localised anthropogenic stressors (e.g. fishing, habitat loss, pollution) (2) | Determines the spatial scale at which fishes interact with potential food sources, and thus how they transfer energy and nutrients across reef habitats (3) |
|  | Diel activity | Nominal | Day, night, both | Mediates the ability of species to avoid predators and competitors (4) | Determines the outcome of bottom-up (i.e. resources consumed) and top-down (i.e. susceptibility to predators) processes (5) |
|  | Group Size | Ordinal | Solitary (sol), pair, small groups (smallg), medium groups (medg), large groups (largeg) | Mediates the susceptibility of fishes to predation and their ability to find food sources, potentially enhancing performance in novel environments (6) | Determines the magnitude of core processes such as herbivory (7) |
|  | Level in water column | Ordinal | Bottom, low, high | Mediates the vulnerability of fishes to the selective impacts of fishing gears (8) | Determines the transport of energy and resources between benthic and pelagic compartments (9) |
|  | Preferred temperature | Continous | Continous, range 15.74°C - 25.52°C | Mediates the response of fishes to shifts in the thermal environment (10) | |
| Morphological | Size class | Ordinal | 0-7 cm (s1), 7.1-15 cm (s2), 15.1-30 cm (s3), 30.1-50 cm (s4), 50.1-80 (s5), > 80 (s6) | Mediates the dispersal and colonization potential of fishes (11), and its often correlated with life history traits that determine fishing vulnerability (12) | Determines trophic interactions (13), and the extent and magnitude of ecological processes such as herbivory (14) |
|  | Body shape | Nominal | Fusiform (fus), Elongated (elo), Compressed (com), Depressed (dep), Box shaped (box), Eel like (eel) | Determines fitness under different hydrodynamic regimes (e.g. wave exposure, currents) (15), and ability to partition complex microhabitats (16) | Often correlated with mobility, and hence influencing transfer on energy and nutrients across reef habitats (3) |

**Table S3. Continued.** List of reef fish functional traits used, including their role in mediating species responses to environmental, biotic, and/or anthropogenic factors (‘Response framework’), and contributions to ecosystem functions (‘Effect framework’).

| **Type** | **Trait** | **Category** | **Levels** | **Response framework** | **Effect framework** |
| --- | --- | --- | --- | --- | --- |
| Morphological | Caudal fin | Nominal | Forked (for), rounded (rou), truncated (tru), pointed (poi) | Determines fitness under different hydrodynamic regimes (e.g. wave exposure, currents) and ability to avoid predators (17) | Often correlated with mobility, and hence influencing transfer on energy and nutrients across reef habitats (3) |
|  | Mouth position | Nominal | Superior (sup), terminal (ter), tubular (tub), elongated (elo), subterminal (subt) | Determine the ability of fishes to capture prey under different environmental constraints (17) | Determines trophic interactions involved in energy fluxes (18) |
| Ecological | Diet | Nominal | Planktivore (pk), mobile invertivore (im), higher carnivore (fc), omnivore (om), herbivore-detritivore (hd), herbivore-macroalgivore (hm), sessile invertivore (is) | Determines the ability of species to expand their geographic ranges (19) | Determines trophic interactions involved in energy fluxes (13, 18) |
|  | Spawning strategy | Nominal | Pelagic, oral, demersal | Determines the sensitivity of fishes to disturbances (19) | |

**Table S4.** Results of multiple generalized linear models testing for differences in fish assemblage structure among depth strata for coral and control assemblages. For simplicity, only species whose abundance differed significantly among depth strata are shown. The likelihood ratio test and associated p-value is indicated.

|  | **Coral** | |  | **Control** | | |
| --- | --- | --- | --- | --- | --- | --- |
| **Species** | **LRT** | **p** |  | **Species** | **LRT** | **p** |
| *Anthias anthias* | 53.70 | 0.001 |  |  |  |  |
| *Bodianus scrofa* | 26.30 | 0.001 |  |  |  |  |
| *Chromis limbata* | 105.56 | 0.001 |  |  |  |  |
| *Coris julis* | 46.09 | 0.001 |  |  |  |  |
| *Lithognathus mormyrus* | 13.96 | 0.03 |  |  |  |  |
| *Scorpaena maderensis* | 15.36 | 0.01 |  |  |  |  |
| *Serranus cabrilla* | 23.57 | 0.001 |  |  |  |  |
| *Similiparma lurida* | 92.86 | 0.001 |  |  |  |  |
| *Sparisoma cretense* | 54.31 | 0.001 |  |  |  |  |
| *Sphyraena sphyraena* | 24.74 | 0.001 |  |  |  |  |
| *Thalassoma pavo* | 98.56 | 0.001 |  |  |  |  |

**Table S5.** Strength of the correlation between individual traits and PCoA axes. For ordinal and nominal traits, the value of the Kruskal-Wallis test indicates relative importance. For continuous traits, the goodness-of-fit (r^2^) of a linear regression model is indicated. Significance levels (p) are reported.

| 1. **PCoA1** | | | | |
| --- | --- | --- | --- | --- |
| **Trait** | **Test** | **Stat** | **Value** | **p** |
| Level water | Kruskal-Wallis | eta2 | 0.798 | 0 |
| Home range | Kruskal-Wallis | eta2 | 0.657 | 0 |
| Group size | Kruskal-Wallis | eta2 | 0.648 | 0 |
| Caudal fin | Kruskal-Wallis | eta2 | 0.63 | 0 |
| Diet | Kruskal-Wallis | eta2 | 0.15 | 0.0419 |
| Body shape | Kruskal-Wallis | eta2 | 0.145 | 0.036 |
| 1. **PCoA2** | | | | |
| **Trait** | **Test** | **Stat** | **Value** | **p** |
| Spawning | Kruskal-Wallis | eta2 | 0.515 | 0 |
| Mouth position | Kruskal-Wallis | eta2 | 0.414 | 0.0001 |
| Body shape | Kruskal-Wallis | eta2 | 0.413 | 0.0002 |
| Diel activity | Kruskal-Wallis | eta2 | 0.272 | 0.0004 |
| Size class | Kruskal-Wallis | eta2 | 0.18 | 0.018 |
| Diet | Kruskal-Wallis | eta2 | 0.176 | 0.0269 |
| Level water | Kruskal-Wallis | eta2 | 0.139 | 0.0106 |
| Preferred temperature | Linear Model | r2 | 0.105 | 0.0166 |
| Home range | Kruskal-Wallis | eta2 | 0.097 | 0.0309 |
| 1. **PCoA3** | | | | |
| **Trait** | **Test** | **Stat** | **Value** | **p** |
| Diet | Kruskal-Wallis | eta2 | 0.552 | 0 |
| Mouth position | Kruskal-Wallis | eta2 | 0.456 | 0 |
| Body shape | Kruskal-Wallis | eta2 | 0.352 | 0.0006 |
| Level water | Kruskal-Wallis | eta2 | 0.255 | 0.0006 |
| Home range | Kruskal-Wallis | eta2 | 0.146 | 0.0088 |
| 1. **PCoA4** | | | | |
| **Trait** | **Test** | **Stat** | **Value** | **p** |
| Caudal fin | Kruskal-Wallis | eta2 | 0.463 | 0 |
| Size class | Kruskal-Wallis | eta2 | 0.208 | 0.0104 |
| Mouth position | Kruskal-Wallis | eta2 | 0.2 | 0.008 |
| Diel activity | Kruskal-Wallis | eta2 | 0.097 | 0.031 |

**Table S6.** Results of generalised linear mixed models (GLMMs) testing for differences in (a) functional richness, (b) functional evenness, and (c) functional divergence among depth strata as a function of the presence of black coral forests (two-way interaction, ‘depth x habitat’). The family error distribution and link function, degrees of freedom (df), chi-squared statistic (Chisq) and associated p-value are shown.

| **(a) FRic** | **Model** | **Family (link)** | **df** | **Chisq** | **p** |
| --- | --- | --- | --- | --- | --- |
|  | Depth | Gamma (“inverse”) | 2 | 1.32 | 0.51 |
|  | Habitat | Gamma (“inverse”) | 1 | 0.01 | 0.90 |
|  | Depth*Habitat | Gamma (“inverse”) | 2 | 5.91 | 0.05 |
|  |  |  |  |  |  |
| **(b) FEve^3** | **Model** | **Family (link)** | **df** | **Chisq** | **p** |
|  | Depth | Gaussian (“identity”) | 2 | 4.05 | 0.13 |
|  | Habitat | Gaussian (“identity”) | 1 | 0.90 | 0.34 |
|  | Depth*Habitat | Gaussian (“identity”) | 2 | 20.55 | **<0.001*** |
|  |  |  |  |  |  |
| **(c) FDiv^3** | **Model** | **Family (link)** | **df** | **Chisq** | **p** |
|  | Depth | Gaussian (“identity”) | 2 | 11.69 | **0.002** |
|  | Habitat | Gaussian (“identity”) | 1 | 0.62 | 0.43 |
|  | Depth*Habitat | Gaussian (“identity”) | 2 | 5.07 | 0.07 |

**Table S7.** Results of generalised linear mixed models (GLMMs) testing for differences in the magnitude of (a) taxonomic and (b) functional dissimilarities among depth strata as a function of the presence of black coral forests (two-way interaction, ‘depth x habitat’), under increasing sensitivity to relative abundances: “q” = 0 (species composition only), “q” = 1 (higher weight on common species), and “q” = 2 (higher weight on dominant species). The family error distribution and link function, degrees of freedom (df), chi-squared statistic (Chisq) and associated p-value are shown.

| **(a) TD** | **"q" = 0** | | | | | **"q" = 1** | | | | | | | | **"q" = 2** | | | | | | | | | | |  |
| --- | --- | --- | --- | --- | --- | --- | --- | --- | --- | --- | --- | --- | --- | --- | --- | --- | --- | --- | --- | --- | --- | --- | --- | --- | --- |
|  | **Model** | **Family (link)** | **df** | **Chisq** | **p** | **Family (link)** | | | **df** | | **Chisq** | | **p** | | | **Family (link)** | | **df** | **Chisq** | | | **p** | |  |  |
|  | Depth | Gaussian (“identity”) | 2 | 550.15 | **<0.001*** | Gaussian (“identity”) | | | 2 | | 570.66 | | **<0.001*** | | | Gaussian (“identity”) | | 2 | 351.30 | | | **<0.001*** | |  |  |
|  | Habitat | Gaussian (“identity”) | 1 | 7.95 | **0.004*** | Gaussian (“identity”) | | | 1 | | 22.08 | | **<0.001*** | | | Gaussian (“identity”) | | 1 | 14.79 | | | **<0.001*** | |  |  |
|  | Depth*Habitat | Gaussian (“identity”) | 2 | 27.15 | **<0.001*** | Gaussian (“identity”) | | | 2 | | 45.67 | | **<0.001*** | | | Gaussian (“identity”) | | 2 | 45.01 | | | **<0.001*** | |  |  |
|  |  |  |  |  |  |  |  |  | |  | |  | | |  | |  | | |  |  | |  | | |
| **(b) FD** | **"q" = 0** | | | | | **sqrt ("q" = 1)** | | | | | | | | **"q" = 2** | | | | | | | | | | |  |
|  | **Model** | **Family (link)** | **df** | **Chisq** | **p** | **Family (link)** | | | **df** | | **Chisq** | | **p** | | | **Family (link)** | | **df** | **Chisq** | | | **p** | |  |  |
|  | Depth | Tweedie (“log”) | 2 | 9.39 | **0.009*** | Tweedie (“log”) | | | 2 | | 302.18 | | **<0.001*** | | | Tweedie (“log”) | | 2 | 301.65 | | | **<0.001*** | |  |  |
|  | Habitat | Tweedie (“log”) | 1 | 24.81 | **<0.001*** | Tweedie (“log”) | | | 1 | | 4.97 | | **0.02*** | | | Tweedie (“log”) | | 1 | 6.87 | | | **0.008*** | |  |  |
|  | Depth*Habitat | Tweedie (“log”) | 2 | 7.89 | **0.01*** | Tweedie (“log”) | | | 2 | | 34.05 | | **<0.001*** | | | Tweedie (“log”) | | 2 | 39.76 | | | **<0.001*** | |  |  |
